# PKM2 activation sensitizes cancer cells to growth inhibition by 2-deoxy-D-glucose

**DOI:** 10.18632/oncotarget.19630

**Published:** 2017-07-26

**Authors:** Sui Seng Tee, Jae Mo Park, Ralph E. Hurd, Kyle R. Brimacombe, Matthew B. Boxer, Tarik F. Massoud, Brian K. Rutt, Daniel M. Spielman

**Affiliations:** ^1^ Department of Radiology, Stanford University, Stanford, CA, USA; ^2^ Applied Sciences Laboratory, GE Healthcare, Menlo Park, CA, USA; ^3^ National Center for Advancing Translational Sciences, NIH, Bethesda, MD, USA; ^4^ NIH Chemical Genomics Center, Bethesda, MD, USA; ^5^ Current/Present address: Memorial Sloan Kettering Cancer Center, New York, NY, USA; ^6^ Advanced Imaging Research Center, University of Texas Southwestern Medical Center, Dallas, TX, USA; ^7^ Department of Radiology, University of Texas Southwestern Medical Center, Dallas, TX, USA

**Keywords:** molecular imaging, metabolic imaging, hyperpolarized MRI, cancer metabolism, PKM2

## Abstract

Cancer metabolism has emerged as an increasingly attractive target for interfering with tumor growth. Small molecule activators of pyruvate kinase isozyme M2 (PKM2) suppress tumor formation but have an unknown effect on established tumors. We demonstrate that TEPP-46, a PKM2 activator, results in increased glucose consumption, providing the rationale for combining PKM2 activators with the toxic glucose analog, 2-deoxy-D-glucose (2-DG). Combination treatment resulted in reduced viability of a range of cell lines in standard cell culture conditions at concentrations of drugs that had no effect when used alone. This effect was replicated *in vivo* on established subcutaneous tumors. We further demonstrated the ability to detect acute metabolic differences in combination treatment using hyperpolarized magnetic resonance spectroscopy (MRS). Combination treated tumors displayed a higher pyruvate to lactate ^13^C-label exchange 2 hr post-treatment. This ability to assess the effect of drugs non-invasively may accelerate the implementation and clinical translation of drugs that target cancer metabolism.

## INTRODUCTION

Cancer cells consume nutrients differently than normal cells, with increased glycolysis present in almost all primary and metastatic cancers [1, 2]. Glycolysis is governed by a number of cytosolic enzymes, with pyruvate kinase (PK) involved in converting phosphoenolpyruvate (PEP) to pyruvate with the simultaneous production of ATP [3, 4]. Alternate splicing of the *PKM* gene results in differential expression of PK isoforms, with PKM2 expressed in cells with high rates of nucleic acid synthesis, including most cancer cells [5-7]. All PK isoforms (with the exception of PKM1) are only active as tetramers and allosteric regulation through stabilization or destabilization of intersubunit contacts determines enzymatic rates [8]. PKM2 has emerged as a new target for cancer therapeutics using both small-molecule inhibitors [9, 10] and activators of the enzyme that enhance tetramerization of PKM2 subunits thereby increasing enzyme activity [11, 12]. PKM2 inhibition has since attracted criticism for potentially promoting tumor growth [13, 14] while PKM2 activators have only been shown to suppress tumor formation when administered immediately after xenograft establishment [11]. Considering the seemingly contradictory data available on PKM2 as a drug target as well as the modest effect of small molecules targeting this enzyme alone, the investigation of additional drugs that synergize PKM2 activation is much needed.

The effective assessment of cancer therapy using non-invasive imaging techniques could guide selection of the most suitable drugs with concomitant benefits to the patient and the healthcare system [15]. In the setting of targeting tumor metabolism as a potential therapeutic target, metabolic imaging has the ability to detect nutrient utilization non-invasively in both pre-clinical studies and in patients. Hyperpolarized magnetic resonance spectroscopy (HP-MRS) is unique in its ability to deliver information on enzyme kinetics. Hyperpolarization describes a number of techniques that are used to enhance the polarization of nuclear spins, with dissolution dynamic nuclear polarization (dDNP) being the mostly widely used to study *in vivo* metabolism [16]. dDNP is based on polarizing nuclear spins in a frozen sample, where microwave irradiation is used to transfer polarization from an organic free radical to an NMR-visible metabolite of interest. Rapid dissolution provides polarized metabolites with greatly enhanced signal-to-noise enabling the detection of metabolic flux *in vivo* [17]. For therapies that directly target glycolysis such as PKM2 activation, the use of hyperpolarized [1-^13^C] pyruvate, a central metabolite in glucose oxidation has the potential to report on the on-target modulation of metabolism *in vivo*.

Here, we investigate the effects of PKM2 activators on cancer cells and *in vivo* tumors, and demonstrate improved inhibition of cancer growth using a PKM2 activator in combination with 2-DG, followed by non-invasive imaging investigation of the metabolic change using hyperpolarized [1-^13^C] pyruvate.

## RESULTS

### PKM2 activation increases aerobic glycolysis with no effect on viability in tumor cell lines

**Figure 1:**
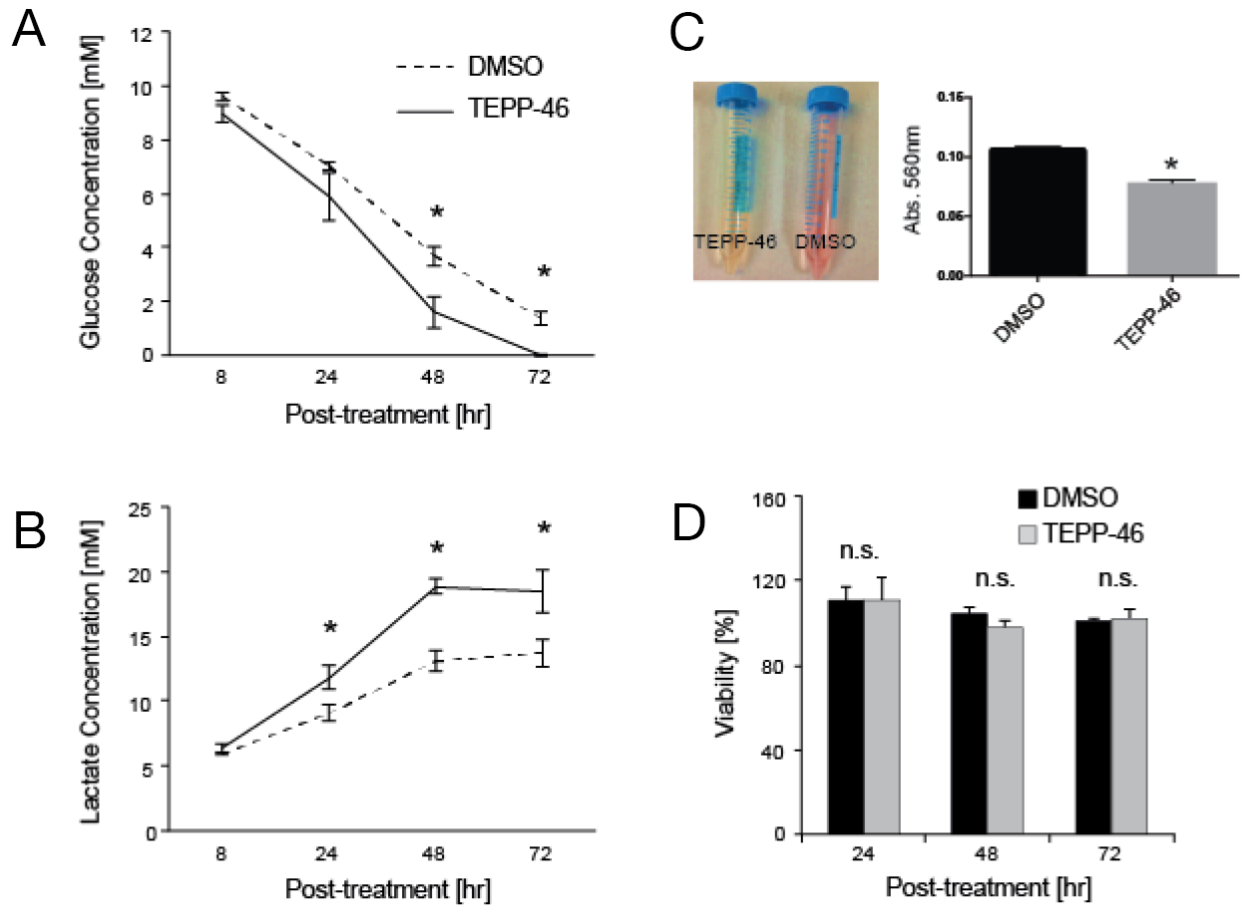
**(A)** Glucose consumption in TEPP46-treated H1299 lung cancer cells significantly increases at 48 hr compared to vehicle-treated cells (1.6 ± 0.6 mM vs. 3.6 ± 0.4 mM, p < 0.05) and **(B)** a concomitant increase of lactate secretion into culture media that is significantly different after 24 hr in TEPP46-treated cells compared to vehicle (11.8 ± 0.9 mM vs. 9.1± 0.6 mM, p < 0.05). This trend continues 48 hr (18.9 ± 0.6 vs. 13.1 ± 0.8 mM, p < 0.01) that results **(C)** a change in the color of phenol red in TEPP-46 treated cells (left panel) that was quantified by absorbance at 516 nm with a significant change in PKM2 activated cells (right panel). **(D)** These changes in metabolism did not result in significantly different changes in viability as measured by MTS oxidation at 24 hr of TEPP46-treated vs DMSO-treated cells (111.1 ± 6.4 vs. 110.3 ± 10.1, p > 0.05) 48 hr (104.5 ± 2.9 vs 97.5 ± 3.2, p > 0.05) or 72 hr (101.1 ± 0.8 vs 102.2 ± 3.7, p > 0.05). All measurements are quoted as averages ± standard deviation of n =3 independent experiments. Statistical significance was determined using student’s t-test, with p < 0.05 deemed significant (^*^) and p < 0.01 (^**^).

We first investigated the metabolic effects of PKM2 activation on H1299 lung cancer cells. While administration of the PKM2 activator, DASA-58, on H1299 cells did not result in increased glucose uptake [11], we observed higher glucose consumption from cell culture media when the same cells were treated with another activator, TEPP-46 that was significantly different from vehicle treatment after 48 hr (1.6 ± 0.6 mM vs. 3.6 ± 0.4 mM, p < 0.05) (Figure 1A). Acidification of cell culture media after 24 hr was also visible as evidenced by the color change of phenol red in culture media (Figure 1B) that can be attributed to increase lactate secretion in TEPP46-treated cells compared to vehicle (11.8 ± 0.9 mM vs. 9.1± 0.6 mM, p < 0.05) (Figure 1C).

Consistent with previously published work [11], there was no significant change in cell viability when treated with TEPP-46 (Figure 1D).

These changes in metabolism motivated the use of hyperpolarized [1-^13^C] pyruvate to investigate if [1-^13^C] lactate would be increased in the presence of TEPP-46. In a suspension of 1 x 10^8^ H1299 cells treated with the drug after 24 hr, we observed a statistically significant increase in the pyruvate to lactate peak ratio (0.0043± 0.0008 vs. 0.0065± 0.0006 A.U., p < 0.05) (Supplementary Figure 1). Lactate dehydrogenase (LDH) enzyme assays on similar treated cells also resulted in higher LDH enzyme activity observed in TEPP46 cells (Supplementary Figure 2), suggesting that increased LDH activity may attribute for both higher lactate secretion into extracellular media as well as driving increased conversion of hyperpolarized lactate after hyperpolarized pyruvate delivery.

Recently, tumor-initiating cells (TICs) derived from a transgenic mouse model of breast cancer have been shown to display a proglycolytic phenotype, with increased lactate production and fewer mitochondria [18]. Forced upregulation of oxidative phosphorylation via administration of the pyruvate dehydrogenase kinase (PDK) inhibitor, sodium dichloroacetate, resulted in a decrease in lactate production that preferentially decreases TIC survival. These results suggest that TICs are exquisitely sensitive to changes in metabolism and may be sensitive to PKM2 activation that had little effect on established cell line viability. Motivated by this observation, we investigated the effects of PKM2 activation on TIC viability. Administration of TEPP-46 has little effect on colony formation of non-tumor initiating cells (NTCs), but reduces the number of TIC colonies (Supplementary Figure 3).

### Combination treatment of TEPP-46 and 2-DG reduces cancer cell viability

**Figure 2:**
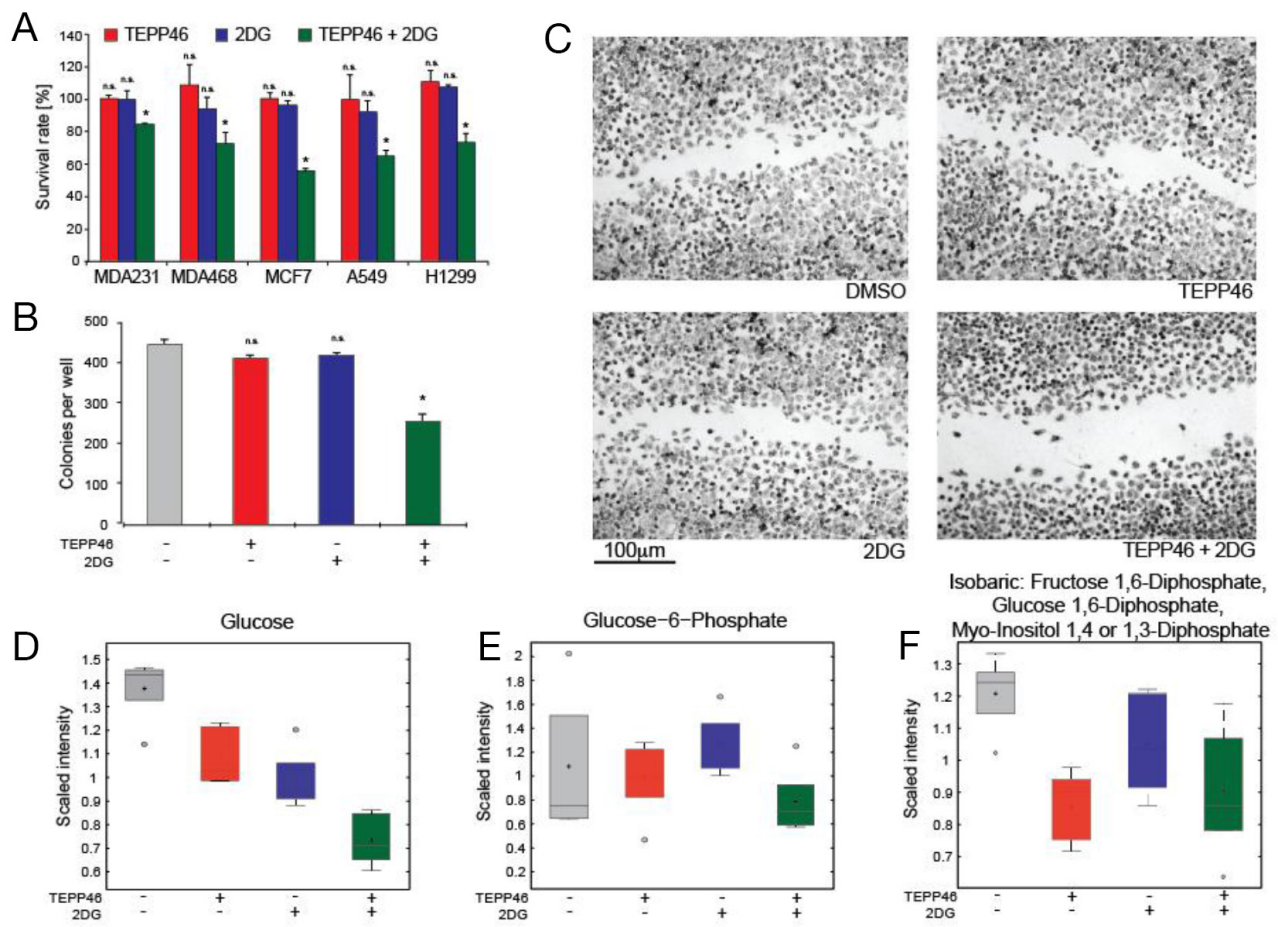
**(A)** Viability of a panel of cell lines (3 breast and 2 lung cancer) assessed by MTT assay after treatment using either TEPP-46, 2-DG or a combination of both. **(B)** Colony forming assay performed on H1299 lung cancer cells seeded at low density in the presence of monotherapy or combination therapy with TEPP-46 and 2-DG. **(C)** Representative images from a scratch-wound assay 48 h after application of either DMSO, TEPP-46, 2-DG or combination treatment. H1299 cells were grown to confluency and a single scratch was introduced to the monolayer before treatments were applied. Metabolic profiling of cells treated with drugs reveal perturbations in glycolytic intermediates, with combination therapy resulting in the lowest levels of glucose **(D)**, glucose-6-phosphate **(E)** and fructose-1,6-bisphosphate **(F)** in cell extracts.

While TEPP-46 reduced viability of TIC’s, we investigated the ability of other metabolic inhibitors that could synergize with PKM2 activation to cause reduced viability in established cancer cell lines. The observation that glucose consumption was increased in TEPP-46 treated cells suggested that combination treatment with 2-DG might result in an increased toxic effect. This glucose analogue is rarely effective as a monotherapy and doses above 63 mg/kg result in hyperglycemia, gastrointestinal bleeding and cardiac abnormalities, thus limiting the utility of 2-DG as a potential chemotherapy [19]. In cell culture studies, TEPP-46 at 30 μM had no effect on cells in standard culture, a result consistent with previous cell culture studies using TEPP-46 at the same concentration [11]. We also observed no significant effect at 1-mM 2-DG in three breast and two lung cancer lines. 2-DG has been shown to induce apoptosis at concentrations of 12 mM [20] but in this study we investigated if TEPP-46 could enhance the inhibitory effect of 2-DG at lower concentrations of the glucose analogue. Indeed, combination treatment resulted in a decrease in viability visible in all five cell lines tested, as measured by the MTT assay (Figure 2A). Additionally, combination treatment also retards the ability of H1299 cells to form colonies as evidenced by the reduction of clonogenic potential when cells were seeded at low densities (Figure 2B). The combination of TEPP-46 and 2-DG also reduced the ability of the same cells to migrate when a confluent monolayer of cells were scratched with a pipette tip (Figure 2C). We performed all cell culture experiments under standard incubation conditions and in the presence of 10-mM D-glucose and 2-mM L-glutamine. These results suggest that combination treatment had a negative effect on cell growth at drug concentrations that had no observable effect individually. Global metabolic profiling was performed to determine the effects of drug treatment on intracellular metabolites. PKM2 activation resulted in lower intracellular pools of glucose, presumably due to increased glycolytic flux through PEP to pyruvate. 2-DG monotherapy also resulted in lower glucose concentrations, while combination therapy yielded the lowest amounts of glucose in the cell (Figure 2D). Similar trends were observed with glucose-6-phosphate (Figure 2E). Fructose-1,6-bisphosphate (Figure 2F) was lowered in both TEPP46 monotherapy as well as combination treatment, alluding to PKM2 activation lowering the levels of glycolytic intermediates.

### Combination treatment reduces growth of established tumors

**Figure 3:**
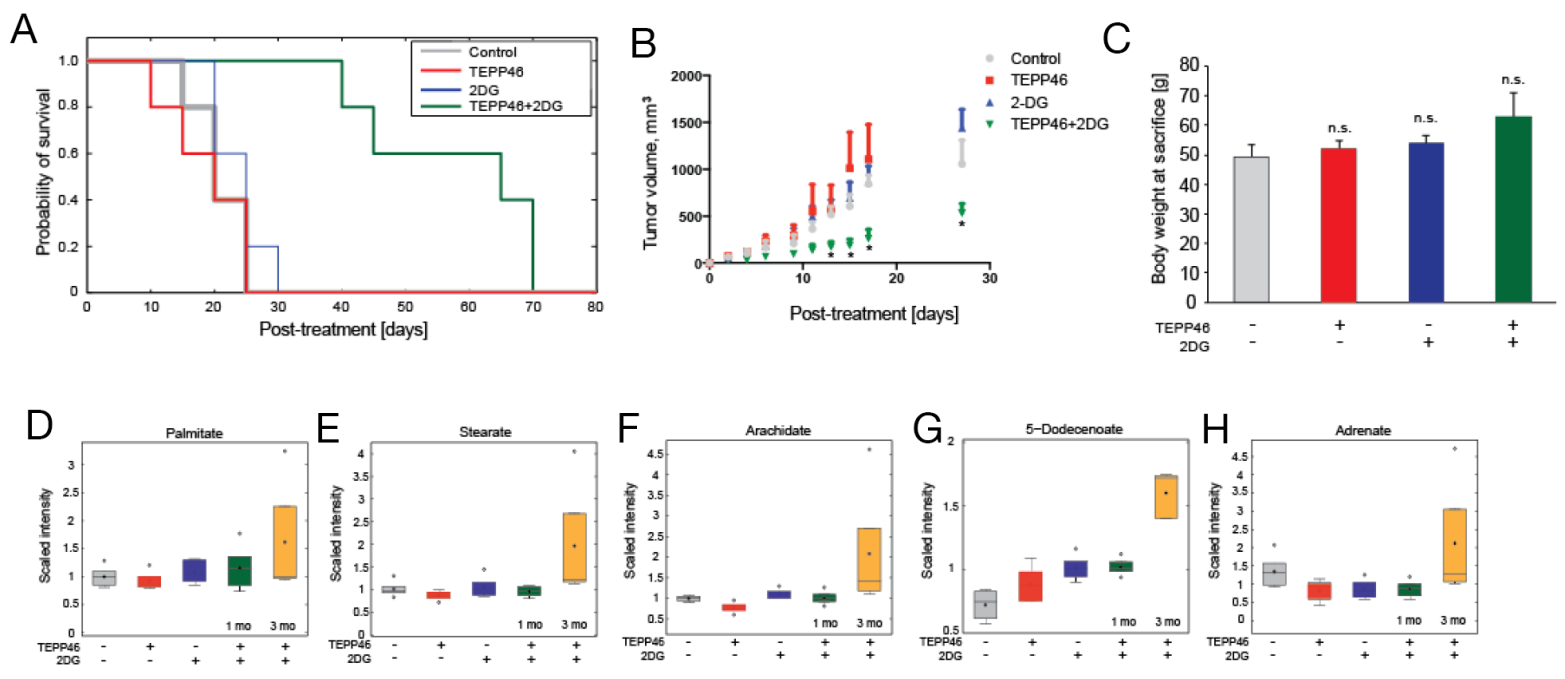
**(A)** Kaplan-Meier curve of four cohorts of mice (n = 5 each) treated with DMSO control, mono-, or combination therapy of TEPP-46 and 2-DG. Treatment was commenced after successful engraftment of xenograft (3-mm caliper measurement, approximately 10 days post-inoculation) and study was terminated as soon as tumors reached terminal diameters as defined by institutional guidelines. All treatments resulted in no significant difference in body weight at sacrifice compared to DMSO control **(B)**. Metabolite profiling revealed higher levels of saturated fatty acids palmitate **(C)**, stearate **(D)** and arachidate **(E)** as well as unsaturated fatty acids 5-dodecenoate **(F)** and adrenate **(G)** in tumors that continued growing 3 months post-combination treatment as compared to tumors that were combination-treated for 1 month.

We proceeded to examine if combination treatment had any effect on established subcutaneous H1299 tumors. TEPP-46 had previously been shown to prevent tumor growth when administered immediately after subcutaneous tumor cell injection [11] but the efficacy of this drug has yet to be tested on established tumors, with the exception of an activator used as competitive inhibitor of an imaging probe [21]. We initiated treatment when any single caliper measurement of a tumor reached 3 mm. The animals were divided into four cohorts (n = 5, each group) and were treated with individual drug, combination treatment, or vehicle control, with the study end-point defined as tumors reaching 1.5 cm^3^. Mice treated with either TEPP-46 or 2-DG alone reached the study end-point at approximately the same time post-treatment as vehicle treated mice (TEPP-46 = 21.8 ± 7 [mean ± std] days, 2-DG = 21.0 ± 5 days and vehicle = 22.6 ± 6 days). In contrast, combination treated animals survived 57.6 ± 12 days (Figure 3A). All treatment regimens resulted in minimal toxicity, with all four treated groups displaying a similar increase in body weight throughout the duration of treatment (Figure 3B). While there was a decrease in tumor growth after combination treatment, all tumors eventually reached the study end-point, suggesting development of adaptive or compensatory mechanisms. To determine the metabolic pathways involved in the development of ‘resistance’ against combination treatment, growth-inhibited tumors at 1 month were compared to ‘resistant’ tumors at 3 months. Decreased transformation of glucose to lipids was previously observed when PKM2 activators were used in cell culture [11]. Therefore we investigated changes in fatty acid metabolism after prolonged treatment with TEPP-46 in mice. Saturated fatty acids (palmitate, stearate and arachidate) (Figure 3C, 3D, 3E) in tumors treated with both TEPP-46 and 2-DG for 3 months were significantly higher than in tumors treated for 1 month. Similarly, unsaturated fatty acid levels (5-dodecenoate and adrenate) (Figure 3F, 3G) were also increased when tumors continued growing for 3 months, suggesting either an increased reliance on fatty acid synthesis or decrease fatty acid oxidation after prolonged treatment with PKM2 activation and 2-DG.

### MR spectroscopic imaging detects metabolic changes in combination-treated tumors

**Figure 4:**
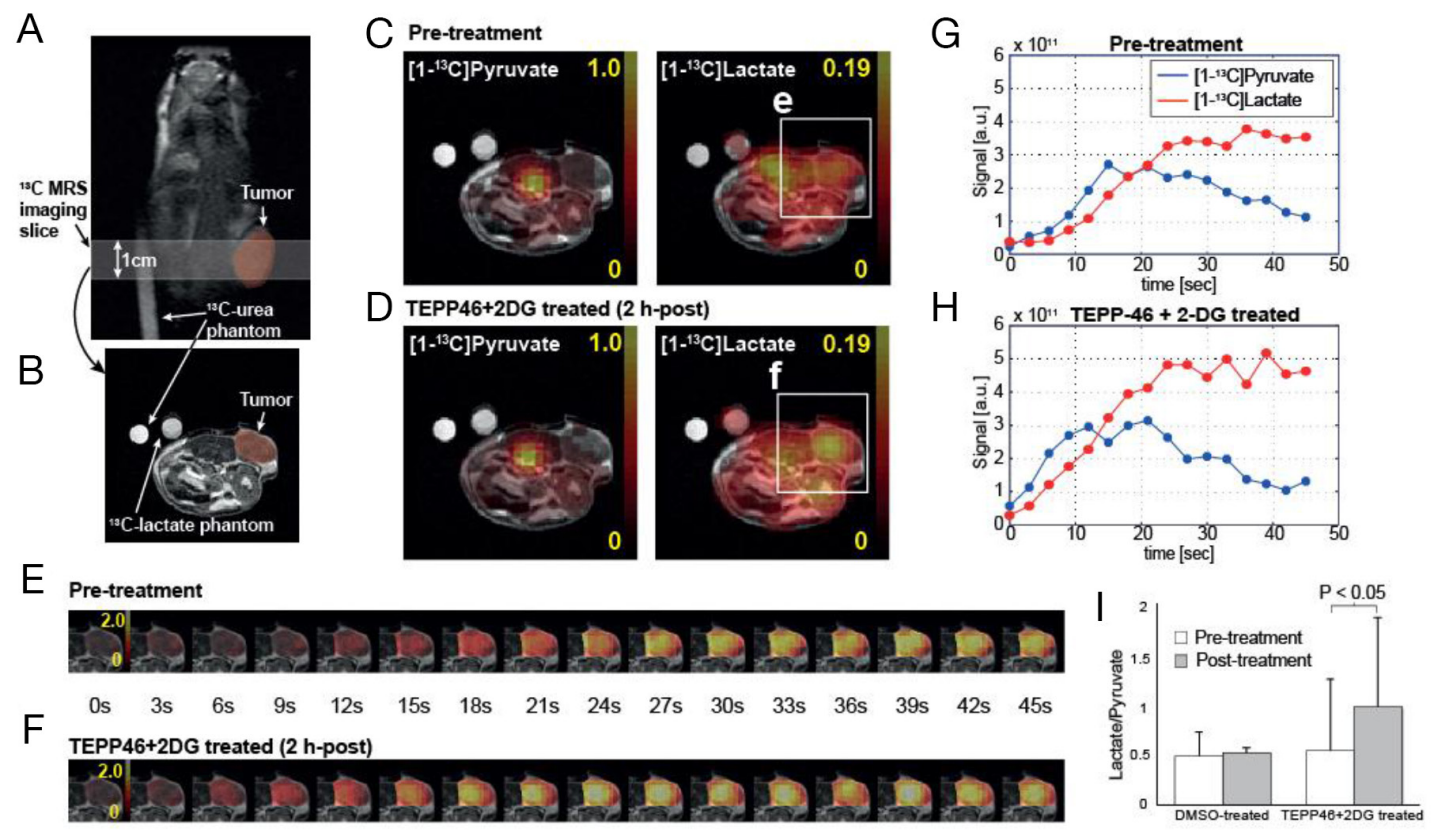
Acute metabolic responses of TEPP-46 and 2-DG combination treatment in subcutaneous tumor measured by hyperpolarized ^13^C MR spectroscopic imaging Dynamic change in pyruvate metabolism was imaged over tumor-bearing slice **(A, B)** every 3 s. Time-averaged ^13^C-pyruvate, ^13^C-lactate, and lactate-to-pyruvate ratio maps **(C, D)** as well as dynamic images **(E, F)** displayed increased lactate labeling from the injected ^13^C-pyruvate in tumors from a representative combination treated mouse as compared to pre-treatment **(G, H)**. No significant change in the lactate-to-pyruvate ratio was observed in DMSO-treated group **(I).**

We then investigated if conversion of hyperpolarized [1-^13^C] pyruvate to [1-^13^C] lactate would be perturbed in the presence of TEPP-46/2-DG combination treatment. The ability to use a clinically relevant imaging modality to visualize changes in metabolism may differentiate tumors that respond to treatment with drugs that change nutrient consumption. Hyperpolarized [1-^13^C] pyruvate was delivered via tail vein in mice with subcutaneous H1299 tumors, before and 2 h after treatment with vehicle or combination therapy. Dynamic change in pyruvate metabolism was imaged over a tumor-bearing slice (Figure 4A, 4B) with a temporal resolution of 3 s immediately following a bolus injection of hyperpolarized [1-^13^C] pyruvate. Time-averaged metabolite maps (Figure 4C, 4D) and dynamic images (Figure 4E–4H) displayed higher ^13^C-lactate conversion of the injected ^13^C-pyruvate in tumors from combination treated mice as compared to the baseline. In particular, metabolite ratios of ^13^C-labeled lactate and pyruvate (lactate/pyruvate), calculated for the tumor region, consistently increased in all mice treated with TEPP-46 and 2-DG together (0.54 ± 0.71 at baseline to 0.98 ± 0.88 at 2 h post-treatment, *P* < 0.05), whereas no significant change in the ratio was observed in DMSO-treated group (*P* > 0.4, Figure 4I). Histological analysis of tumors immediately after imaging studies revealed no significant changes in morphology (Supplementary Figure 4)

## DISCUSSION

In this study we demonstrate for the first time activation of PKM2 using a small molecule, which results in an increased consumption of glucose and secretion of lactate in cancer cells. These metabolic alterations were not observed using another PKM2 activator [22] and the reverse was actually seen in yet another study where PKM2 activation resulted in decreased lactate secretion [11]. However, our use of a different activator, with a different structure than reported previously might account for these conflicting results. Regardless, to the best of our knowledge, all PKM2 activators have little effect on cancer cell proliferation under conventional cell culture conditions. In contrast to immortalized cancer cells, TICs are growth inhibited under conventional culture conditions upon PKM2 activation. Our observations are consistent with several other studies [23-25] that have also noted the increased sensitivity of these stem-like cells to metabolic perturbation.

We hypothesized that increased glucose consumption as a result of PKM2 activation afforded an opportunity to sensitize the anti-proliferative effects of this class of drugs. Glucose transport in cancer cells is predominantly facilitated by GLUT1 [26] and this transporter has also been reported to facilitate uptake of the non-metabolizable analogue of glucose, 2-DG [27]. 2-DG has long been studied as a cancer therapy, with clinical trials reported almost two decades ago [28]. However, owing to unacceptable toxicity at high doses, further clinical trials of 2-DG were halted [29]. We observed a decrease in viability across lung and breast cancer cell lines when PKM2 activation was combined with 2-DG and justifies future experiments to investigate if this effect is generalizable across a larger panel of cancer cells. Interestingly, metabolite profiling revealed low concentrations of glucose and related sugar phosphates in combination-treated cells, supporting the notion that acceleration of PKM2 activity using TEPP-46 results in increased glucose metabolism. Although we did not perform experiments to determine that minimum 2-DG concentration that might still be effective for treatment, future experiments to determine this dose will be beneficial. Additionally, other combinations that target other metabolic targets may also sensitize cells to PKM2 activation. For example, the glutaminase inhibitor, CB-839 has been postulated to prevent entry of exogenous glutamine into the TCA cycle [30] and when combined with PKM2 activation that accelerates glucose backbones to lactate conversion, might result in a chronic lack of carbons for anabolic reactions.

The anti-growth effects of combination treatment were also observed in a subcutaneous lung cancer xenograft model. In contrast to previous studies [11, 31] using small molecules activators of PKM2 *in vivo*, we commenced treatment after the appearance of palpable tumors. While there was a significant decrease in the growth of combination-treated tumors, eventually all animals succumbed. This acquisition of ‘resistance’ is commonly seen in therapies targeted towards cancer metabolism and is attributed to activation of alternative pathways to obtain energy [32]. Specifically, disruption of glucose metabolism has already been shown to promote fatty acid synthesis [33] as well as glutamine metabolism [34]. While we did not elucidate the signaling pathways that led to ‘resistance’ in combination therapy, a more thorough understanding of the mechanisms that enable metabolic plasticity will be crucial for the future development of therapies that target tumor nutrient consumption. An understanding of the relevant metabolic pathways that compensate for PKM2 activation in the long term may also provide for new methods to detect ‘resistance’ to therapy using molecular imaging modalities such as HP-MRS.

There are conflicting studies on the role of PKM2 in cancer, with both inhibitors and activators having anti-tumor effects [35]. These conflicting studies are attributed PKM2 having multiple roles, both metabolic and non-metabolic, that may have differential effects in distinct cell types [36]. Considering this level of complexity, we are of the opinion that PKM2 modulation alone is unlikely to impact tumor growth. To the best of our knowledge, this study represents the first demonstration of combining PKM2 modulation with a proven anti-cancer drug to slow cancer progression. It is entirely possible that targeting metabolism via PKM2 may only be viable in a subset of tumors. This highlights the importance of having reliable methods to stratify patients that might potentially respond and subsequently, technologies to assess effective treatment.

In this study we demonstrate the feasibility of assessing PKM2-targeted therapy using hyperpolarized MR – an imaging modality currently trialed in patients in multiple centers worldwide. The first clinical trials using hyperpolarized ^13^C-pyruvate have demonstrated good safety profiles [37, 38] of the imaging agent as well as the ability to detect lactate labeling in regions of tumors. A large number of pre-clinical studies have also demonstrated the ability of hyperpolarized pyruvate to detect positive treatment response. To date, almost all of these studies have linked a lower rate of apparent pyruvate-to-lactate conversion with treatment efficacy, regardless of the type of treatment used for cancer. These include standard chemotherapy [39, 40], targeted therapies [41, 42], ionizing radiation [43, 44] as well as focused ultrasound ablation [45]. The only exception was reported by Lodi and colleagues, where treatment of prostate cancer cells, PC3, with a MEK inhibitor resulted in an increase of hyperpolarized pyruvate to lactate conversion [46]. In this study, we have also reported a similar observation, where a potential cancer therapeutic has increased conversion to lactate. We have not investigated the biological changes that facilitate this observation *in vivo*, but in cell culture with TEPP46 alone, we did observe in increase in LDH activity. Previous studies have also shown that LDH activity is directly linked to the availability of co-factor NADH [39]. PKM2 activation increases flux through glycolysis, resulting in increased conversion of NAD^+^ to NADH. It is possible that LDH activity is then accelerated to compensate for increased NAD^+^ consumption to maintain redox homeostasis in cancer cells. Activation of LDH by phosphorylation at Tyr10 has already been postulated to maintain co-factor levels [47] and could be the same modification that facilitates increased pyruvate-to-lactate conversion seen in our hyperpolarized experiment. Investigations on the levels of co-factors as well as post-translational modifications of enzymes should be performed to elucidate the underlying mechanism of increased hyperpolarized lactate production. Interpretation of the parameters that define ‘successful’ treatment also needs to be scrutinized especially as this modality is scheduled for assessing treatment response in a number of clinical trials in then near future. Although *in vivo* HP-MRS were performed 2 hr after treatment due to logistical difficulties of transporting animals back to the scanner after longer treatment regimes, it will be interesting to investigate if the same increase in ^13^C-lactate is observed.

Recently, several radioactive tracers have been developed to query the status of PKM2 expression *in vivo* using PET imaging. By ^11^C-radiolabelling the PKM2 activator, DASA-23, Witney and colleagues were able to obtain images from orthotopic, patient-derived glioblastoma xenografts that were very specifically localized to regions of the tumor that expressed PKM2 [21]. Beinat and colleagues have also synthesized an ^18^F-labeled version of the same molecule. While radiolabelled PKM2 activators are ideal as a companion diagnostic to stratify patients that might respond to PKM2 activation, we postulate that hyperpolarized ^13^C-pyruvate could then be used to monitor effective therapy after treatment. Although we did not longitudinally assess the prognostic value of increased lactate-to-pyruvate ratio and inhibition of tumor growth, the acute effects of combination therapy have demonstrated the possibility of using metabolic imaging to assess metabolic therapy of tumors.

In conclusion, we describe a novel metabolic consequence of PKM2 activation. By harnessing increased glucose consumption following combination use of 2-DG and TEPP-46, we demonstrate inhibition of cancer growth *in vitro* and *in vivo*. Importantly, augmenting the anti-tumor effects of 2-DG by combination with another drug is a valuable translational therapeutic strategy, since a major drawback of 2-DG in clinical trials has been dose-limiting toxicities. We have also shown the ability to assess on-target inhibition of combination treatment using hyperpolarized MRS. As more drugs targeting glycolysis are developed, our studies confirm the utility of metabolic imaging as a non-invasive method to probe the specificity and potentially, the efficacy of therapy.

## MATERIALS AND METHODS

### Cell culture, viability measures and drug treatments *in vitro*

All cell culture was performed under standard culture conditions of 37°C and 5% CO_2_. A549 and H1299 cells were grown in RPMI media (Gibco, CA) supplemented with 5% fetal calf serum (FCS) and penicillin/streptomycin. MDA-MB-231, 436 and MCF-7 cells were grown in DMEM media (Gibco, CA) supplemented with 5% FCS and penicillin/streptomycin.

Viability assays were performed using CellTiter 96 Aqueous One Solution (Promega, CA) according the manufacturer’s instructions. After seeding cells at 5,000 per well, treatments were administered 24 hr before performing viability assays.

Scratch assays were performed on H1299 cells grown to confluence in a 6-well plate. A P10 pipette tip was used to create a scratch on the monolayer, before culture media was substituted with either DMSO or TEPP-46 containing media. Cells were allowed to grow for an additional 24 hr before pictures were taken using a 20X objective on a phase-contrast microscope.

Colony proliferation assay was performed on 6-well plates. After seeding cells at a density of 600 cells per well, cells were allowed to grow for approximately 10 days with or without treatment as indicated. After this time, a 50:50 solution of methylene blue:methanol was used to fix and stain cells. Brightfield microscope pictures were obtained from 3 different regions in each well for each treatment condition performed in triplicate. Colonies were then counted with ImageJ (NIH, Bethesda, MD) after manual threshold selection.

TEPP46 was a generous gift from Dr. Matthew Boxer (NIH National Center for Advancing Translational Science). Stock solutions of 30 mM were prepared in DMSO. All dilutions to working solution were performed in appropriate cell culture media. 2-DG (Sigma, CA) was prepared to working solutions in appropriate cell culture media.

### Xenograft establishment and drug treatments *in vivo*

3-4 week old female nu/nu mice were purchased from Charles River Laboratories (CA). 1 × 10^7^ H1299 freshly trypsinized cells in 100-μl volume of 50:50 Matrigel (BD Biosciences, CA):PBS were injected subcutaneously in the right flank. Caliper measurements and body weights were measured every 2 days.

TEPP-46 (50 mg/kg) and 2-DG (180 mg/kg) were diluted in 40% beta-hydroxycyclopropane (Sigma, CA). Treatments were administered intra-peritoneally daily for five days with a two-day break in between, for the entirety of the experiment. All experimental procedures were conducted in accordance with the US National Institutes of Health guidelines and were approved by the Stanford University Institutional Animal Care and Use Committee.

### Global metabolic profiling

Metabolite profiling was performed on both cell culture samples as well as tumor fragments. Cells were trypsinized after indicated treatments, centrifuged, washed in PBS and rapidly frozen in liquid nitrogen. For tumors, fragments of approximately 200 mg were excised, rinsed in PBS and rapidly frozen in liquid nitrogen. The metabolic profiling platform we used was the same as methods published previously [48].

### Hyperpolarized ^13^C MR spectroscopic imaging

H1299-implanted mice were imaged using hyperpolarized MRS 8-12 weeks after the subcutaneous cell injections. The progression of the tumors was volumetrically monitored daily to achieve comparable tumor sizes (∼500 mm^3^). All mice were imaged with hyperpolarized ^13^C MRS imaging. Animals were anesthetized with 1-1.5 % isoflurane in oxygen (∼1.5 L/min), had a tail vein cannula placed, and were then positioned at the center of a clinical GE 3T MR scanner and a high performance insert gradient coil (G_max_ = 500 mT/m, Slew rate_max_ = 1865 mT/m/ms). A custom-made ^13^C-^1^H dual-channel coil was used for both RF excitation and signal acquisition. Vital signs were monitored throughout the experiments, and the body temperature was maintained ∼36.5^o^C using a temperature-regulated air heater. The homogeneity of the B_0_ field over the tumor region was manually optimized with a point-resolved spectroscopy sequence using the linear shim currents. Single-shot spiral chemical shift imaging (CSI) with spectral undersampling (field of view = 43.5 × 43.5 mm^2^, 6 mm slick thickness, matrix size = 16 × 16, variable flip angle RF excitation, temporal resolution = 3 s, 16 time points, 32 echoes, spectral bandwidth = 280 Hz) was used for dynamic metabolic imaging of hyperpolarized [1-^13^C] pyruvate and its products. The ^13^C spectra were acquired 6 s after the start of a bolus injection (300 μL of 80-mM pyruvate over ∼20 s) at baseline and 2 h after the treatments. For anatomical references, both T_2_-weighted fast spin-echo and contrast-enhanced T_1_-weighted spin-echo images were acquired. Integrated signal for individual metabolite peak in time-averaged spectrum was used to calculate ^13^C-labeled lactate-to-pyruvate ratios. Paired student t-test was performed to evaluate statistical significance of treatment effects (α = 0.05).

## SUPPLEMENTARY MATERIALS FIGURES


