# Supplementary material for: PKM2 activation sensitizes cancer cells to growth inhibition by 2-deoxy-D-glucose
Source: Oncotarget. 2017 Jul 26;8(53):90959–68. doi: 10.18632/oncotarget.19630 (PMC5710897; doi:10.18632/oncotarget.19630)
Supplement: Supplementary file 1 [file oncotarget-08-90959-s001.pdf]

PKM2 activation sensitizes cancer cells to growth inhibition by 2-deoxy-D-glucose

SUPPLEMENTARY MATERIALS

Tumor processing for histological analysis

Xenograft tumors were dissected and fixed overnight in 10% neutral-buffered formalin. The tissue was then dehydrated and embedded in paraffin, sectioned and stained with hemotoxylin-eosin. The Stanford University Department of Comparative Medicine Histology Lab performed all procedures.

Hyperpolarized [1-13C] pyruvate spectroscopy in cell suspensions

H1299 cells were treated with either DMSO or TEPP46 for 24 hr. Immediately before spectroscopy, cells were trypsinized and resuspended in media. This was followed by an injection of 2 ml of 14-mM hyperpolarized [1-^{13}C] pyruvate solution, which had been polarized using HyperSense dynamic nuclear polarizer. All MR measurements were performed using 3-T GE clinical MR scanner and a custom-built surface coil ($\varnothing_{in}$ = 28 mm). Dynamic free induction decay spectroscopic sequence (spectral width = 5,000 Hz, spectral points = 2048) with hard RF pulse excitations (pulse width = 40µs, nominal flip angle = 10°) was used to acquire spectra with 3 s of temporal resolution (T_{acq} = 4:00 min).

Lactate dehydrogenase (LDH) enzyme asaay

H1299 cells were treated for 24 hr in 30µM TEPP46 and immediately washed in ice-cold PBS. Cells were lysed with RIPA buffer (Thermo Scientific, CA) with protease and phosphatase inhibitors added to 1 x concentration (Thermo Scientific, CA). 10 µg of total protein in the lysate was added for LDH enzyme assay, in a 96-well plate format containing 1mM sodium pyruvate, 0.22 mM NADH and 200mM Tris HCl buffer at pH 7.3. Absorbance of NADH was read at 340nm.

Tumor harvest, dissociation, isolation and flow cytometry

Tumors from MMTV-*Wnt-1* FVB/NJ (002934; Jackson Laboratory, Bar Harbor, ME) female transgenic mice were harvested and processed according to methods described previously [1, 2]. According to established protocols, tumor initiating cells (TICs) had expression of CD49f^{high} and Epcam^{low}, markers commonly found in mammary basal stem/progenitor cells while non-tumor initiating cells (NTCs) were CD49f^{low} and Epcam^{high}, with markers associated with luminal differentiation

3-dimensional cancer spheroid assay

FACS-purified MMTV-Wnt1 tumor cells were resuspended in culture medium and plated on top of solidified Matrigel (BD Bioscience) as described previously [18]. The culture medium was changed every 2 days and only colonies measuring 50 µm in diameter were counted.

REFERENCES

1. Cho RW, Wang X, Diehn M, Shedden K, Chen GY, Sherlock G, Gurney A, Lewicki J, Clarke MF. Isolation and molecular characterization of cancer stem cells in MMTV-Wnt-1 murine breast tumors. Stem Cells. 2008; 26:364-71.
2. Diehn M, Cho RW, Lobo NA, Kalisky T, Dorie MJ, Kulp AN, Qian D, Lam JS, Ailles LE, Wong M, Joshua B, Kaplan MJ, Wapnir I, Dirbas FM, et al. Association of reactive oxygen species levels and radioresistance in cancer stem cells. Nature. 2009; 458:780-3.

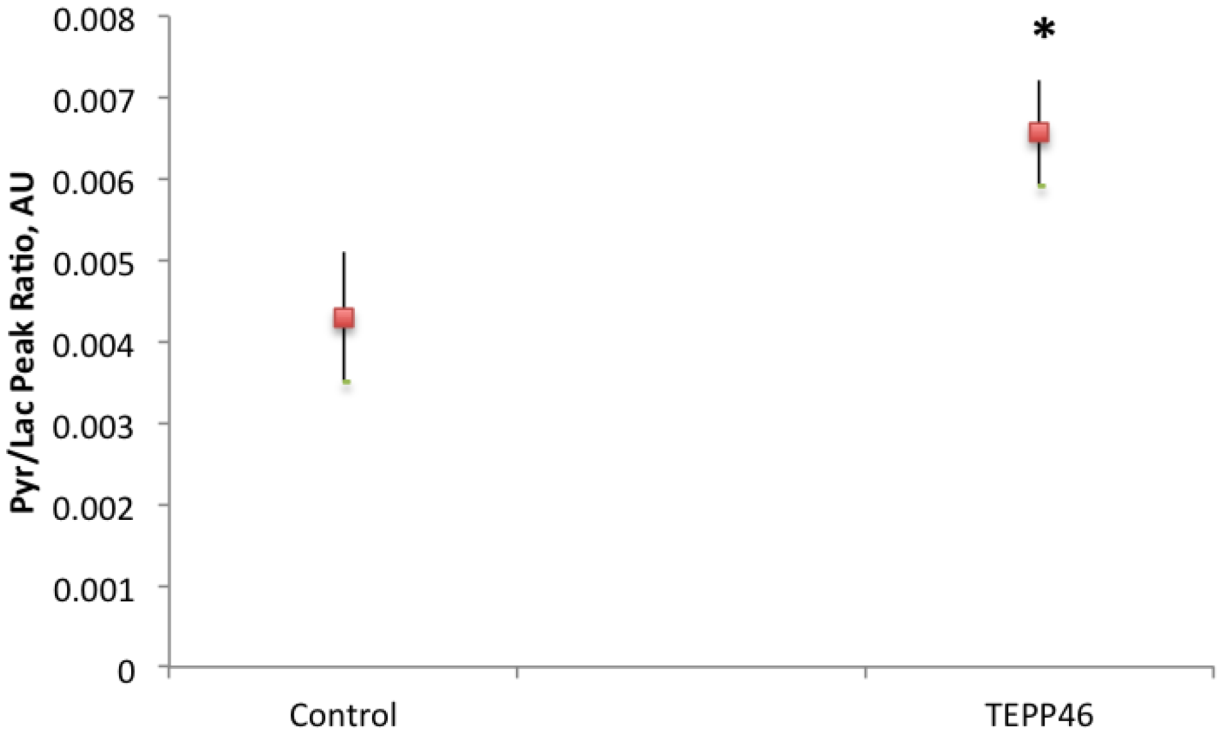

Supplementary Figure 1: Quantification of pyruvate peak over lactate peak ratio in H1299 cells treated with 30uM TEPP46. A total of 1 x 10^8 cells were trypsinized and resuspended. 2 ml of 14-mM hyperpolarized [1-^{13}C] pyruvate solution, which had been polarized using HyperSense dynamic nuclear polarizer. All MR measurements were performed using 3-T GE clinical MR scanner and a custom-built surface coil ($_{in}$ = 28 mm). Dynamic free induction decay spectroscopic sequence (spectral width = 5,000 Hz, spectral points = 2048) with hard RF pulse excitations (pulse width = 40µs, nominal flip angle = 10°) was used to acquire spectra with 3 s of temporal resolution (T_{acq} = 4:00 min).

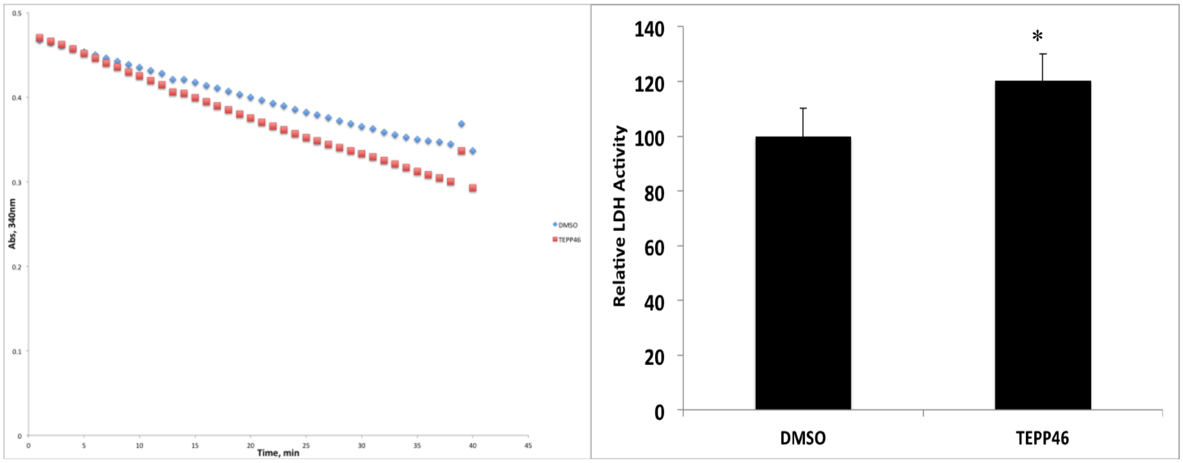

Supplementary Figure 2: (Left) Kinetics of NADH absorbance over time of 50ug of total protein lysate from H1299 cells treated with either DMSO or TEPP46 for 24 hr, in the presence of 1mM sodium pyruvate, 0.22 mM NADH and 200mM Tris HCl buffer at pH 7.3.(Right) Quantification of relative LDH activity compared to untreated H1299 cells was statistically significant (p<0.05, n=3 each condition).

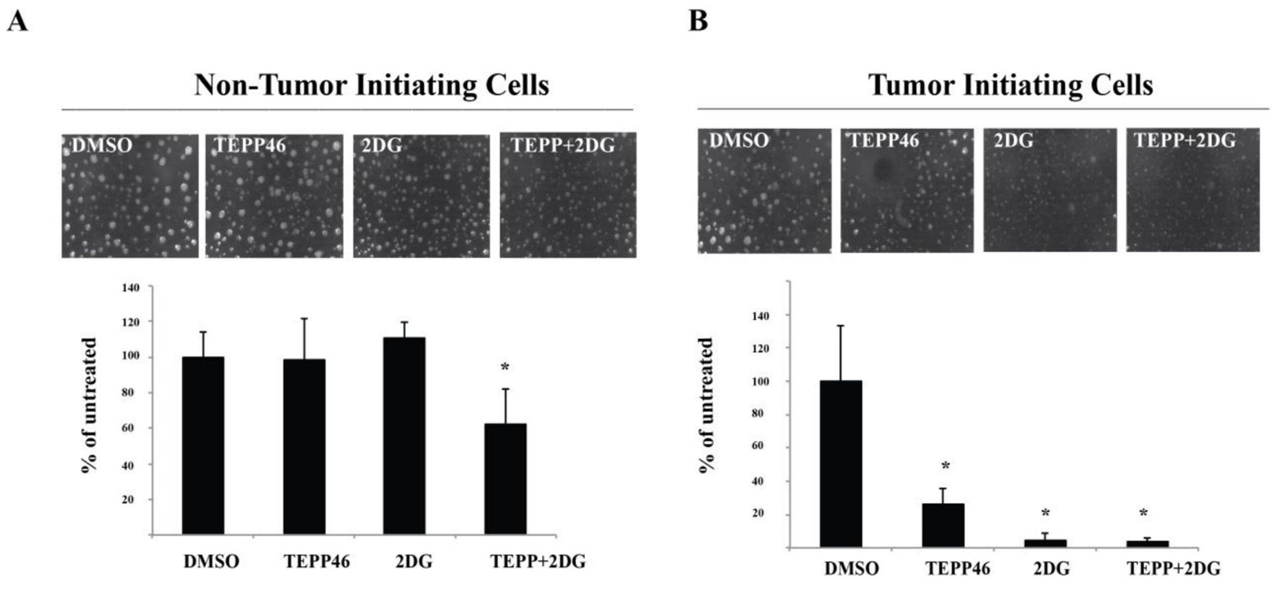

Supplementary Figure 3: (A) Representative images from wells of non-tumor initiating cells (NTCs) treated with either vehicle (DMSO), TEPP-46, 2-DG or a combination of TEPP-46 and 2-DG. Quantification of colony formation was performed by applying manual thresholds and normalized against untreated wells. **(B)** Representative images from wells if tumor-initiating cells (TICs) with similar treatment conditions as NTCs.

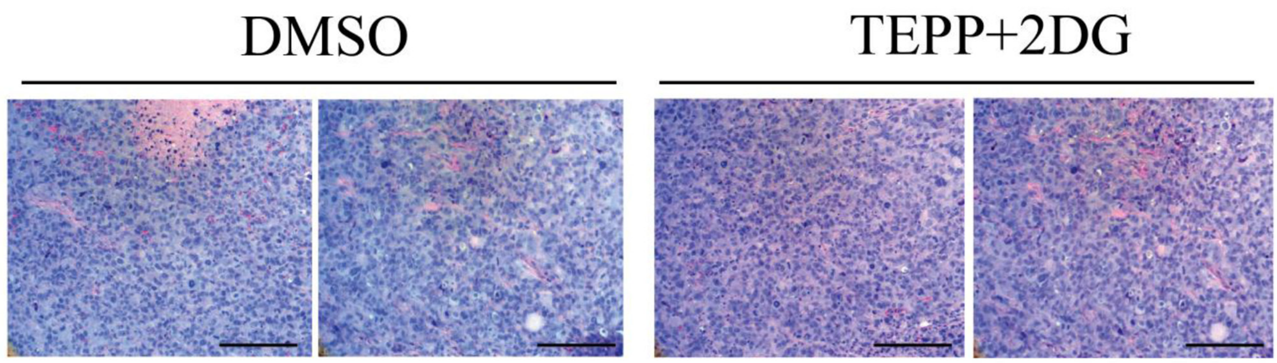

Supplementary Figure 4: H&E stained sections of tumors treated with either DMSO or combination of 2-DG and TEPP-46 for 2 h. Scale bar represents 1mm.
